# A Retrospective Cohort Study on The Impact of the Enhanced Recovery After Surgery with Safe Brain Initiative on Total Knee Arthroplasty Outcomes in Türkiye

**DOI:** 10.4274/TJAR.2026.252248

**Published:** 2026-02-09

**Authors:** Berna Çalışkan, Şahan Dağlar, Mine Gürsaç Çelik, Başak Ceyda Meco, Finn M. Radtke

**Affiliations:** 1 University of Health Sciences Türkiye, İstanbul Haseki Training and Research Hospital, Clinic of Anaesthesiology and Reanimation, İstanbul, Türkiye; 2 University of Health Sciences Türkiye, İstanbul Haseki Training and Research Hospital, Clinic of Orthopaedics and Traumatology, İstanbul, Türkiye; 3 University of Health Sciences Türkiye, Bakırköy Dr. Sadi Konuk Training and Research Hospital, Clinic of Anaesthesiology and Reanimation, İstanbul, Türkiye; 4 Ankara University Faculty of Medicine, Department of Anaesthesia and Intensive Care, Ankara, Türkiye; 5 University of Southern Denmark, Nykøbing Falster Hospital, Department of Anaesthesia and Intensive Care, Odense, Denmark

**Keywords:** Enhanced recovery after surgery, safe brain initiative, pain management, length of stay, total knee arthroplasty

## Abstract

**Objective:**

Enhanced recovery after surgery (ERAS) protocols are recognised for improving postoperative outcomes. Integrating structured prehabilitation with the safe brain initiative (SBI) may further enhance these benefits. This study evaluated the impact of an ERAS-SBI programme on postoperative recovery and analgesic requirements in patients undergoing total knee arthroplasty (TKA).

**Methods:**

This retrospective single-centre cohort study included adults classified as American Society of Anesthesiologists I-III who underwent elective TKA at a tertiary-care teaching hospital. Outcomes of patients managed with the ERAS-SBI programme (n = 138; December 2023-2024) were compared with those of patients treated prior to programme implementation (n = 66; December 2022-2023). The primary outcome was the length of hospital stay. Secondary outcomes included timing of postoperative discharge and cumulative rescue opioid analgesia at 24 and 48 hours.

**Results:**

The ERAS-SBI group had a significantly shorter hospital stay than the pre-ERAS-SBI group (*P* < 0.001). The time to postoperative discharge was also reduced (*P* < 0.001). Rescue opioid analgesia consumption at 24 and 48 hours was significantly lower in the ERAS-SBI group (*P* < 0.001 for both comparisons). Perioperative anaemia and blood transfusion rates were reduced in the ERAS-SBI group (*P*=0.007 and *P*=0.003, respectively).

**Conclusion:**

Implementing an ERAS-SBI pathway, incorporating a dedicated prehabilitation-focused ERAS outpatient clinic, is associated with shorter hospitalisation and reduced postoperative analgesic requirements following TKA. These findings support the role of enhanced multidisciplinary perioperative optimisation in improving clinical outcomes.

**Main Points**

• Enhanced recovery after surgery (ERAS) programs improve surgical outcomes by standardising multimodal, evidence-based perioperative care.

• Patient-centred prehabilitation and multidisciplinary coordination are considered important; however, their implementation is inconsistent across many institutions.

• This study is among the first to evaluate an integrated ERAS and safe brain initiative (SBI) protocol, which begins in a dedicated preoperative ERAS outpatient clinic and continues in a postoperative ERAS recovery room.

• The implementation of the ERAS-SBI protocol in total knee arthroplasty resulted in optimised patient recovery, enhanced pain management, and improved resource utilisation.

## Introduction

Initially designed to enhance perioperative patient care in colorectal surgery, enhanced recovery after surgery (ERAS) protocols have also been successfully applied to orthopaedic surgery.^1^ Studies examining the adaptation of ERAS protocols for total joint arthroplasty have demonstrated significant reductions in hospital length of stay (LOS), mortality rates, complications, and the need for blood transfusions.^2^ However, there is an ongoing interest in improving the steps defined by the ERAS Society and implementing multidisciplinary coordination in practice.^1^ ERAS results are improved more effectively through better organisation of prehabilitation strategies than by altering intraoperative techniques or analgesia methods.^3^ To achieve this goal, a specially designed outpatient clinic that coordinates predefined, multidisciplinary steps could be transformative.

Previous ERAS literature regarded short LOS and early postoperative rehabilitation as desirable outcomes. However, to accelerate recovery, the practitioner should minimise the patient’s physical and psychological stress, primarily by ensuring effective pain management.^4^ However, the same anaesthesia and analgesia plan may not result in the same pain perception or the same rehabilitation success.^5^ Personalised care could be the missing piece that completes the entire puzzle, making personalised care suitable for implementation in ERAS protocols such as the safe brain initiative (SBI), which aims to improve perceptions of brain health by reducing anxiety and mental stress.^6^

In this study, we investigated the impact of implementing an ERAS-SBI programme on postoperative outcomes following total knee arthroplasty (TKA), while maintaining consistency in surgical technique and anaesthesia management. The primary outcome was length of hospital stay (LOS). Secondary outcomes included postoperative discharge time, requirements for rescue opioid analgesia within 48 hours, perioperative anaemia and blood transfusion rates, and postoperative cognitive recovery parameters, assessed using ERAS-SBI tools.

## Methods

### Study Design and Patient Selection

This retrospective single-centre cohort study was conducted at a tertiary-care teaching hospital following approval from the University of Health Sciences Türkiye, İstanbul Haseki Training and Research Hospital, Clinical Research Ethics Committee (approval no.: 50-2025, date: 09.04.2025). We studied American Society of Anesthesiologists (ASA) I-III patients who underwent TKA in the orthopaedic operating room. Patients were excluded if they withdrew from surgery, were unable to complete the ERAS outpatient clinic protocols, or were unable to cooperate with medical staff during rehabilitation. Two consecutive time-defined patient cohorts were evaluated: the ERAS-SBI group (patients treated after implementation of the ERAS and SBI; December 2023-December 2024; n = 138) and the pre-ERAS-SBI group (patients treated prior to programme implementation; December 2022-December 2023; n = 66). Data from both groups were collected from the hospital system and from follow-up interviews conducted in the wards. Additional prehabilitation information for the ERAS-SBI cohort was available from the ERAS outpatient clinic system. Finally, we obtained data for all 138 patients in the ERAS-SBI-treated period and for all 66 patients in the pre-ERAS-SBI period.

### ERAS Outpatient Clinic and Orthopaedic Ward

From December 2023 onward, patients at the orthopaedic outpatient clinic with an indication for TKA who were scheduled for surgery were referred to the ERAS outpatient clinic. Patients requiring treatment for anaemia were directed to the anaemia outpatient clinic, where, based on the timing of their surgery and the severity of their anaemia, they initiated intravenous (IV) iron therapy or oral iron replacement therapy. Sarcopenia was evaluated using the hand-grip test, and, if necessary, patients were referred to the nutrition unit for nutritional planning or supplementation. Smokers were referred to a smoking cessation support unit at least four weeks prior to their surgery to assist them in quitting.

Finally, all patients were referred preoperatively to the physiotherapy unit, where they were instructed in range-of-motion and muscle-strengthening exercises for three weeks.

Patients admitted to the surgical ward were placed in a dedicated ERAS room under the supervision of a trained ERAS nurse. At 22:00 on the night before surgery, patients were provided with a light, high-protein snack (such as yoghurt and eggs) in accordance with ERAS nutritional principles to maintain perioperative energy and protein balance. On the morning of surgery, clear fluids (up to 400 mL water or tea) were permitted at 06:00. ERAS surgical care principles included no routine use of tourniquets or drains and administration of tranexamic acid (1 g IV).

### ERAS-SBI Protocol

All patients in the ERAS-SBI group were managed using the SBI patient-centred precision care approach. Patient-reported outcomes — including pain, anxiety, stress, thirst, nausea, vomiting, and overall well-being — were evaluated using the standard SBI questionnaire. These parameters were re-assessed both upon arrival in the post-anesthesia care unit and at discharge.^6^

**Figure 1:**
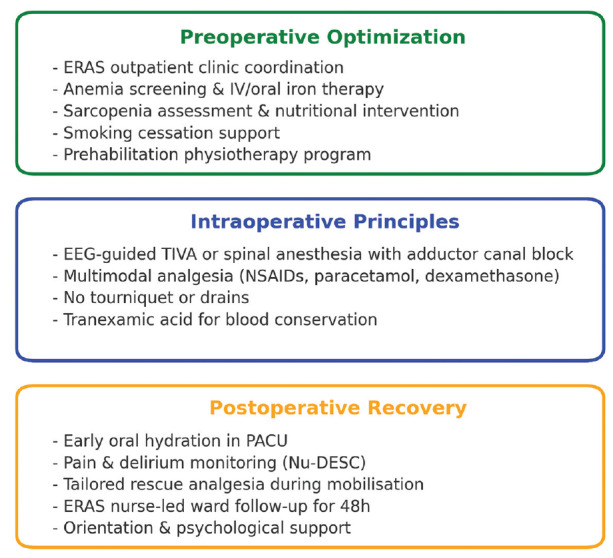
ERAS-SBI protocol key components. NU-DESC, nursing delirium screening scale; ERAS, enhanced recovery after surgery; SBI, safe brain initiative;  IV, intravenous; EEG, electroencephalography; TIVA, total intravenous anesthesia; NSAIDs, non-steroidal anti-inflammatory drugs; PACU, post-anesthesia care unit.

Additionally, cognitive status and delirium were assessed using the Nursing Delirium Screening Scale (NU-DESC), a validated tool scored 0-10, where a score of ≥2 indicates delirium. If the patient opted for general anaesthesia, we administered total intravenous anaesthesia (TIVA) under electroencephalographic (EEG) guidance. For those who consented to regional anaesthesia, we administered spinal anaesthesia (17.5 mg of heavy Marcaine and 10 mcg of fentanyl) and a postoperative adductor canal block (20 mL of 0.375% bupivacaine) for pain relief in both scenarios. The multimodal analgesia plan, started intraoperatively in the surgical ward, included dexamethasone 8 mg (IV), the nonsteroidal anti-inflammatory drug (tenoxicam 1x1), paracetamol (1 g 3x1), and tramadol (1 mg kg^-1^, maximum 400 mg/day) as rescue therapy. Rescue opioids were administered if the numerical rating scale score was ≥4 at rest or ≥6 during mobilisation. Ondansetron (4 mg) was used for prophylaxis against postoperative nausea and vomiting (PONV). In the recovery room, a physician supervised the administration of 200 mL of water and assessed pain control and delirium scores. The ERAS nurse and anaesthesiologist jointly monitored patients for 48 hours postoperatively (Figure 1).

### Non-ERAS-SBI Protocol

The non-ERAS-SBI group differs from the ERAS-SBI group in its affiliation with the ERAS outpatient clinic and in the preoperative preparation it receives. In this group, the general anaesthesia protocol used sevoflurane rather than EEG-guided TIVA and other applications of SBI because early oral hydration was not available. However, the multimodal analgesia plan, which included a postoperative adductor canal block, remained the same for both time periods.

Because the same senior anaesthesiologist managed all perioperative care, the analgesia protocol used after ERAS-SBI adoption was identical to that routinely applied before ERAS-SBI implementation. All procedures were performed by the same senior orthopaedic surgical team using a medial parapatellar approach with a cemented prosthesis.

### Clinical Data and Outcome Variables

For both groups, the following were recorded: demographic variables [age, gender, body mass index (BMI), ASA score], the presence of preoperative anaemia (defined as Hb 12 g dL^-1^ on the day of surgery), and the amount of perioperative blood transfusion.

The primary outcome was LOS, defined as the number of days from admission to discharge. Secondary outcomes included postoperative discharge time, defined as hours from arrival in the ward after surgery until criteria-based discharge home, and total rescue opioid analgesia at 24 and 48 hours.

Moreover, for the ERAS-SBI group, we documented additional variables, including sarcopenia, frailty, anxiety score, smoking status, preoperative sleep time (on the day before surgery), and the presence of preoperative and postoperative delirium (screened by NU-DESC).

### Statistical Analysis

The descriptive statistics for the data included the mean, standard deviation, median, minimum, maximum, frequencies, and ratios. The distribution of the variables was assessed using the Shapiro-Wilk test. The Mann-Whitney U test was employed to analyse independent quantitative data that were not normally distributed. For the analysis of independent qualitative data, the chi-square test was used. The analyses were conducted using IBM SPSS Statistics for Windows, version 28.0 (IBM Corp., Armonk, NY, USA).

## Results

The age and sex distributions did not differ significantly between the ERAS-SBI and non-ERAS-SBI groups (Table 1). BMI was significantly higher in the ERAS-SBI cohort (35.8±6.2 kg m²^-1^ vs. 32.2±5.6 kg m²^-1^, *P* < 0.001, r = 0.28). Most patients in both groups were classified as ASA II; however, the pre-ERAS-SBI group did not include any ASA I patients (*P* < 0.001, Table 1).

**Figure 2:**
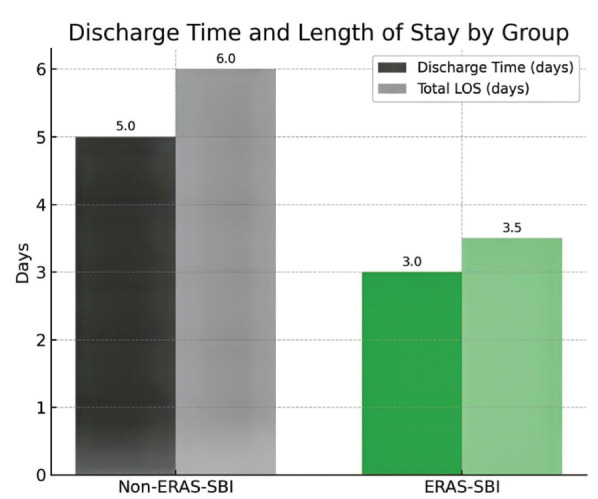
Comparison of discharge time and total length of hospital stay. ERAS, enhanced recovery after surgery; SBI, safe brain initiative; LOS, length of stay.

The LOS was significantly shorter in the ERAS-SBI group [median 3 (3-4) days] than in the non-ERAS-SBI group [median 5 (4-6) days; *P* < 0.001, r = 0.63] (Figure 2). Likewise, postoperative discharge time was reduced [median 28 (24-36) hours vs 45 (36-54) hours; *P* < 0.001, r = 0.58].

Total rescue opioid analgesic use was significantly lower in the ERAS-SBI group at both postoperative time points (*P* < 0.001; Table 2). In the first 24 hours, 81.2% of ERAS-SBI patients required no rescue opioids, compared with 25.8% of non-ERAS-SBI patients. A similar pattern was observed between 24 and 48 hours (81.2% and 21.2%, respectively).

The distribution of anaesthetic techniques differed significantly between groups (Table 2). General anaesthesia combined with a peripheral nerve block was used more frequently in the ERAS-SBI group than in the non-ERAS-SBI group (61.8% vs. 31.9%;* P* < 0.001). In contrast, spinal anaesthesia combined with peripheral nerve block was more common in the non-ERAS-SBI group (46.9% vs. 18.7%; *P* < 0.001). Rates of postoperative adductor canal block were similar between groups (80.5% vs. 78.8; *P*=0.76), as were rates of epidural analgesia (27.0% vs. 21.1; *P*=0.43) (Table 2).

**Figure 3:**
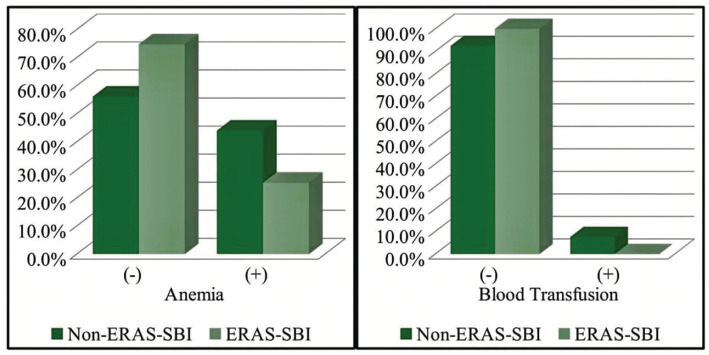
Comparison of anemia and perioperative blood transfusion of the same study population. ERAS, enhanced recovery after surgery; SBI, safe brain initiative.

Preoperative anaemia was significantly less frequent in the ERAS-SBI group compared with the non-ERAS-SBI group (25.4% vs. 43.9%, *P*=0.007, odds ratio: 0.43; 95% confidence interval: 0.23-0.81). Perioperative blood transfusions occurred in 7.6% of patients in the non-ERAS-SBI group, whereas none were recorded in the ERAS-SBI group; Figure 3). Postoperative complication rates were comparable between groups (9.4% vs. 10.6; *P*=0.790), and there was no statistically significant difference in intensive care unit admission rates.

Prehabilitation-related variables (frailty, sarcopenia, anxiety, smoking status, and sleep duration) were comprehensively recorded only in the ERAS-SBI cohort (Table 3), revealing prevalences of sarcopenia (34.8%) and median frailty and anxiety scores of 5 and 3, respectively prior to surgery.

## Discussion

This study is the first to report the clinical consequences of the ERAS-SBI protocol, achieved by organising an ERAS outpatient clinic and implementing the SBI approach. A key finding of this study is the marked reductions in both discharge time and total LOS following the implementation of the ERAS-SBI protocol. These outcomes are consistent with published ERAS literature showing that coordinated perioperative pathways improve postoperative outcomes following arthroplasty.

The primary objective of ERAS protocols is to establish a system that reduces both the LOS and overall healthcare costs.^7^ To achieve this, the individual components of ERAS have been evaluated with a focus on promoting early mobilisation and reducing postoperative complications.^8^ Although our shorter hospital stay aligns with ERAS literature, addressing variations in the application of accepted ERAS protocols can help adapt protocols to clinical differences.^1^ While ERAS programs are widely recognised for their potential to accelerate recovery, the strategic integration of prehabilitation, personalised care via the SBI, and multidisciplinary coordination can further enhance these benefits. Shortening the LOS is not merely a metric of efficiency — it is a direct indicator of enhanced patient recovery, reduced risk of complications associated with prolonged hospitalisation, and more efficient use of healthcare resources. From a health economics standpoint, our LOS reduction translates into substantial savings. Adding secondary benefits — such as reduced transfusions, lower opioid consumption, and increased surgical throughput — raises the potential of these systems without compromising safety. Importantly, our data showed that complication rates and intensive care unit admissions did not increase despite earlier discharge, indicating that the gains in efficiency were accompanied by maintained safety standards. In our cohort, the median LOS decreased significantly compared with the non-ERAS-SBI period, despite surgical techniques and multimodal analgesia regimens remaining unchanged because the same surgeon and anaesthesiologists managed both cohorts. This underlines that the organisational and patient-centred modifications — not procedural innovations — were the drivers of improvement. The ERAS outpatient clinic structure facilitated the early detection and targeted management of modifiable preoperative risks such as anaemia, sarcopenia, smoking, and preoperative anxiety, all of which are known to delay mobilisation and discharge. Moreover, personalised counselling and expectation-setting primed patients psychologically for early discharge, amplifying the protocol’s effect. However, interpretation requires caution because the ERAS-SBI cohort had fewer ASA III patients and exhibited differences in BMI, which may partially explain faster recovery and lower transfusion requirements. Studies on ERAS have shown that postoperative opioid use remains high, even among ERAS patients.^9^ Thus, the effectiveness of our analgesic management could be due to the implementation of SBI as a patient-centred precision-care approach. It led us to revisit a lesser-discussed aspect of ERAS: the role of patient-dependent variables, including physical and mental prehabilitation, in redefining personalized pain perception. Furthermore, the presence of a dedicated nurse for ERAS-SBI patients may have significantly influenced this outcome. This ERAS-SBI nurse was trained to minimise unnecessary opioid use and to actively assess the patients’ pain levels. This assessment included not only subjective evaluations using a visual analogue scale (VAS) but also observational assessments during postoperative mobilisation, which may have further contributed to a reduced need for analgesics. Although intraoperative anaesthetic techniques differed between groups, postoperative analgesia was standardised throughout both study periods, using the same multimodal regimen of IV paracetamol, tenoxicam, dexamethasone, and a single-shot adductor canal block with identical local anaesthetic volumes and concentrations. Furthermore, administration of rescue analgesia was protocol-driven and based on predefined VAS thresholds, thereby reducing the likelihood that lower opioid use reflected altered prescribing behaviour rather than pain-related need. Despite this consistency, the ERAS-SBI group required less postoperative rescue analgesia, suggesting that non-pharmacological factors inherent to the SBI framework—such as structured communication, expectation management, and continuous patient-centred assessment—may have influenced pain outcomes.

Recommended interventions by the ERAS Society for perioperative care in knee replacement surgery begin with preoperative information and counselling to the patient.^1^ In our ERAS protocol, the outpatient clinic educates patients and implements these strategies to foster expectations of early discharge. Although the initial part of the ERAS recommendations is supported by a low level of evidence, the literature suggests that setting patient expectations can positively impact LOS.^10^ While this finding highlights the influence of psychological strategies for which evidence is limited, incorporating the SBI approach into the ERAS protocol may amplify its positive impact on patient expectations. The preoperative recommendations from the SBI project emphasise the importance of preventing oral dehydration and minimising prolonged fasting periods, such as those in ERAS protocols. Instead, we encouraged oral liquid intake in the recovery room, regardless of anaesthesia type, to ensure early resumption of oral intake.

Additionally, SBI focuses on enhancing patient orientation and maintaining a regular day-night rhythm.^6^ Therefore, we assess these patients in the recovery room for pain and delirium; during this assessment we tell them the time of day and remind them of the therapeutic benefits of early mobilisation when pain is absent and of early discharge from the hospital. These measures are designed to reduce perioperative anxiety and delirium and, ultimately, to facilitate earlier patient discharge, as demonstrated by decreased LOS and shorter discharge times after our ERAS-SBI protocol.

As pain-free early mobilisation is the most critical factor for early discharge, perioperative analgesia management is the most influential factor in these protocols. Peripheral nerve blocks are generally preferred to epidural or IV patient-controlled analgesia (PCA) for managing postoperative pain after primary arthroplasty.^11^ This preference arises from a lower incidence of neurological side effects, reduced nausea and vomiting, and earlier mobilisation. Given the innervation of the knee joint, either a femoral nerve block or an adductor canal block can be used; however, the adductor canal block is recommended to minimise motor block associated with a femoral nerve block, which is undesirable for early mobilisation.^12^ In line with these recommendations, we implemented an adductor canal block as the peripheral nerve block for patients in our ERAS-SBI protocol. Epidural PCA was usually considered when complete pain relief was necessary for 48 hours because of patient-specific considerations, such as anatomical or psychological factors. We did not use an adductor canal catheter because it had limited cost-effectiveness.^13^ In postoperative pain management following TKA, different nerve blocks and their combinations are increasingly utilised, with protocols prioritising early mobilisation.^14^ Thus, future studies may identify a more effective block strategy that aligns better with ERAS protocols than the currently used adductor canal block.

The correction of anaemia during the preoperative optimisation phase is increasingly regarded as a crucial strategic change. Preoperative anaemia is linked to higher transfusion requirements and associated with various perioperative complications and health issues, such as an increased risk of cardiac events and cancer recurrence.^15, 16^ IV iron therapy is particularly effective during the perioperative period due to its rapid onset of action. Therapeutic approaches in this field are continually evolving, particularly with respect to dosing strategies and administration methods.^17^ Establishing a dedicated anaemia clinic that focuses on the diagnosis, treatment, and follow-up of patients with anaemia could significantly improve the quality of perioperative care. Our institution benefits from such a clinic, which has become a key component of our ERAS protocol. In our cohort, in which the mean patient age exceeds 65 years, the prevalence of preoperative anaemia on the day of surgery decreased from 45% to 25%. Importantly, none of the patients in the ERAS-SBI group required a perioperative blood transfusion. The remaining 25% of anaemia cases were likely due to factors such as patient noncompliance or challenges coordinating treatment at the anaemia clinic with surgical scheduling. As the protocol continues to develop, eliminating preoperative anaemia remains a primary objective for future phases.

Sarcopenia is commonly identified in the geriatric population and is known to adversely affect early postoperative mobilisation following major joint surgeries, thereby contributing to prolonged hospital stays.^18^ For this reason, patients identified with sarcopenia during ERAS clinic evaluations were referred to a separate outpatient nutrition clinic for further assessment and follow-up. As a result, nutritional support was planned for 34.8% of these patients. However, no reassessment was performed prior to surgery, and the effectiveness of prehabilitation interventions for anaemia and sarcopenia could not be measured quantitatively, except for those reflected in the overall results.

When examining our PONV rates, we considered the 5.1% incidence a favourable outcome, particularly given that our population primarily consisted of elderly female patients undergoing arthroplasty, groups traditionally associated with a higher risk of PONV.^19^ Several factors likely contributed to this low incidence, including the routine administration of dexamethasone (8 mg) and ondansetron, as recommended, and a reduced postoperative opioid requirement.^20^ Additionally, shortening fasting durations and facilitating oral fluid intake within the first hour postoperatively, as part of the SBI approach, may have further supported this outcome.^21^

While certain intraoperative elements consistent with ERAS recommendations were already standard practice, the ERAS-SBI programme introduced structured prehabilitation, multidisciplinary coordination, and the SBI framework as new components. To further enhance the ERAS-SBI protocol, future efforts may explore additional intraoperative modifications, such as avoiding the use of bone cement.^2^ Despite recent literature examining the key components of ERAS protocols and demonstrating success in their primary objectives, there may nonetheless be a need to redefine those objectives beyond strategies that merely optimise known outcomes.^22, 23^ Future studies could place greater emphasis on prehabilitation and patient-centred care by developing a multidisciplinary approach and organisation similar to initiatives such as the Safe Brain Project.^24^

Although our ERAS-SBI protocol showed improvement over our standard care protocol, we must recognise that further enhancements are possible, particularly through the adaptation of hospital facilities to accommodate this system. Although postoperative discharge time was significantly shorter in the ERAS-SBI group, we found that LOS nevertheless remained longer than three days owing to one key reason. Specifically, hospital facilities—such as the number of beds, availability of operating rooms, and access to prosthetic materials—may significantly influence the ability to achieve rapid discharge, rather than this ability depending solely on patient recovery protocols. For surgical specialities that rely on equipment, such as orthopaedics, streamlining these processes could enable even greater gains from ERAS-SBI implementation.

### Study Limitations

This study represents the initial evaluation of the ERAS-SBI protocol within our institution; therefore, several limitations should be considered. The retrospective, single-centre design introduces an inherent risk of selection bias and limits generalisability. Additionally, although the surgical and anaesthetic techniques remained consistent between groups, baseline differences in the ERAS-SBI cohort, such as higher BMI and variations in ASA classification, may have influenced outcomes independently of the protocol implementation. Because multivariate or propensity-based adjustment was not performed, the potential confounding effects of these variables cannot be excluded, and causal inferences must be interpreted cautiously. Furthermore, institutional workflow constraints and incomplete data in the non-ERAS-SBI group precluded one-to-one matching across all variables, which may have introduced additional bias.

Another limitation relates to the incomplete assessment of postoperative functional recovery. For example, the exact time to first mobilisation could not be recorded, although it is an essential marker of ERAS success, particularly in TKA. Likewise, long-term cognitive trajectories were not captured. Although the SBI framework emphasizes perioperative brain health, postoperative cognitive dysfunction beyond the acute period remains to be studied. Patient-reported outcome measures (PROMs) were not incorporated into this preliminary implementation phase, despite their value in capturing patient-centred recovery.

Future research should build on these findings using prospective, preferably multicentre, study designs with multivariable adjustment to better assess confounding. We plan to incorporate routine PROMs, early mobilisation metrics, and long-term cognitive outcomes into the ERAS-SBI outpatient pathway to obtain a more comprehensive assessment of functional and patient-centred outcomes.

## Conclusion

The implementation of the ERAS-SBI protocol represents an important step towards optimising perioperative care in TKA, particularly in elderly populations, who are vulnerable to perioperative cognitive dysfunction. Enhanced prehabilitation, guided by a single ERAS clinic across multiple specialised outpatient clinics, could lead to further improvement in postoperative care within the ERAS-SBI protocol, thereby resembling a system of gears that functions through distinct yet harmoniously integrated mechanisms. Although accelerated recovery is often reflected in outcomes such as a shortened LOS, our ultimate goal extends beyond this. Rather than focusing solely on the joint, we aim for comprehensive physical and psychological recovery of the patient as a whole. When recovery is conceptualised in this broader, patient-centred manner, future studies, guided by more nuanced and multidimensional assessments, may achieve even more meaningful outcomes.

## Ethics

**Ethics Committee Approval:** This retrospective single-centre cohort study was conducted at a tertiary-care teaching hospital following approval from the University of Health Sciences Türkiye, İstanbul Haseki Training and Research Hospital, Clinical Research Ethics Committee (approval no.: 50-2025, date: 09.04.2025).

**Informed Consent:** Retrospective study.

## Figures and Tables

**Table 1. Demographic Variables:** 

-	**Non-ERAS-SBI (n = 66)**	**ERAS-SBI (n = 138)**	***P* value**
**Age (years)**	65.8±8.2 (66.0)	65.1±8.4 (66.0)	0.760^m^
**Sex**	-	-	0.255ᵡ²
Female	54 (81.8%)	103 (74.6%)	-
Male	12 (18.2%)	35 (25.4%)	-
**BMI (kg m²^-1^)**	32.2±5.6 (32.2)	35.8±6.2 (35.2)	<0.001^m^
**ASA physical status**	-	-	<0.001ᵡ²
I	0 (0.0%)	29 (21.0%)	-
II	38 (57.6%)	81 (58.7%)	-
III	28 (42.4%)	28 (20.3%)	-

Data are presented as mean ± standard deviation (median) or n (%)

^m^: Mann-Whitney U test, ᵡ²: Chi-square test (Fisher’s exact test where appropriate)

BMI, body mass index; ASA, American Society of Anesthesiologists; ERAS, enhanced recovery after surgery; SBI, safe brain initiative

**Table 2. Perioperative Anesthesia and Analgesia Management of the Study Groups:** 

**Postoperative rescue analgesia (0-24 h)**	**Non-ERAS-SBI (n = 66)**	**ERAS-SBI (n = 138)**	***P* value**
0 doses	17 (25.8%)	112 (81.2%)	<0.001ᵡ²
1 dose	2 (3.0%)	3 (2.2%)	0.721ᵡ²
2 doses	23 (34.8%)	4 (2.9%)	<0.001ᵡ²
3 doses	24 (36.4%)	19 (13.8%)	<0.001ᵡ²
**Postoperative rescue analgesia (24-48 h)**			
0 doses	14 (21.2%)	112 (81.2%)	<0.001ᵡ²
1 dose	2 (3.0%)	1 (0.7%)	0.276ᵡ²
2 doses	27 (40.9%)	3 (2.2%)	<0.001ᵡ²
3 doses	23 (34.8%)	22 (15.9%)	<0.001ᵡ²
**Type of anaesthesia**			
General anaesthesia + PNB	21 (31.9%)	86 (61.8%)	<0.001ᵡ²
Spinal anaesthesia + PNB	31 (46.9%)	26 (18.7%)	<0.001ᵡ²
Combined spinal-epidural	13 (19.6%)	14 (10.1%)	0.067ᵡ²
General anaesthesia + epidural	1 (1.5%)	13 (9.4%)	0.031ᵡ²
**Postoperative ICU admission**			
No	64 (97.0%)	138 (100%)	0.104ᵡ²
Yes	2 (3.0%)	0 (0.0%)	-
**Postoperative complications**			
No	59 (89.4%)	125 (90.6%)	0.790ᵡ²
Yes	7 (10.6%)	13 (9.4%)	-

Data are presented as n (%)ᵡ²: Chi-square test (Fisher’s exact test applied where appropriate)

ICU, intensive care unit; ERAS, enhanced recovery after surgery; SBI, safe brain initiative; PNB, peripheral nerve block (adductor canal block)

**Table 3. Additional Variables Specific to ERAS-SBI Group:** 

-	**Mean ± SD / n-% median (range)**
**Smoking status** No use Quit (3 weeks before surgery) Resume smoking	- 123-89.1% 7-5.1% 8-5.8 %
Anemia outpatient clinic IV iron therapy Oral iron theraphy	27-19.57 % 3-2.17 % 5-3.67 %
Frailty score (1-9)	5.0±0.1, 5.0 (4.0-6.0)
Sarcopenia (handgrip test positive) (nutrition outpatient clinic supplement)	48-34.8 %
Preoperative anxiety score (0-10)	2.9±0.9, 3.0 (1.0-6.0)
Preoperative delirium (NU-DESC +) Postoperative delirium (NU-DESC +)	0.0 % 0.0 %
Sleep time, the day before surgery (h)	6.0±0.9, 6 (5.0-10.0)
**PONV at the first 24h** (+) (-)	- 7-5.07 % 131-94.9 %

ERAS, enhanced recovery after surgery; SBI, safe brain initiative; PONV, postoperative nausea and vomiting; SD, standard deviation
